# Bystanders to Bias: Witnessing Gendered Microaggressions Affects Men’s and Women’s Outcomes in STEM Small Group Contexts

**DOI:** 10.3390/bs15020215

**Published:** 2025-02-14

**Authors:** Nadia Vossoughi, Logan C. Burley, Ryan P. Foley, Lorelle A. Meadows, Denise Sekaquaptewa

**Affiliations:** 1 Department of Psychology, Grinnell College, Grinnell, IA 50112, USA; 2 Department of Psychology, Queen’s University, Kingston, ON K7L 3N6, Canada; 22prg1@queensu.ca; 3 Department of Psychology, University of Michigan, Ann Arbor, MI 48109, USA; 4 Department of Psychology and Human Factors, Michigan Technological University, Houghton, MI 49931, USA; lameadows@mtu.edu

**Keywords:** gender bias, stereotypes, STEM, gender

## Abstract

We tested whether merely witnessing gendered microaggressions affects group work experiences among male and female undergraduate computer science and engineering students. Across three experiments (*N* = 753), we randomly assigned computer science and engineering students to witness microaggressions targeting female students, or control interactions, using a video manipulation. Witnessing microaggressions—compared to the control—resulted in heightened gender-specific stereotyping concerns, with women being concerned about appearing incompetent and men being concerned with appearing sexist. For both women and men, witnessing microaggressions resulted in decreased enthusiasm for participating in group work. Moreover, for women, the relationship between decreased enthusiasm and witnessing microaggressions was partially mediated by increased concerns about being stereotyped as incompetent. Across the experiments, mixed results emerged regarding the effect of witnessing microaggressions on the recall of engineering content in the video. This research extends previous work focused on personally experiencing microaggressions to merely witnessing them, showing that positivity toward anticipated group work is diminished for both women and men when they see peers engaging in microaggressions.

## 1. Introduction

Gender bias and underrepresentation have been pervasive issues experienced by women in most science, technology, engineering, and mathematics (STEM) fields for decades (Long, 2001; NASEM, 2007; National Science Foundation, 1994, 2021), demonstrating both the gravity of these issues as well as their persistence. Research demonstrates that much of the gender bias women experience is subtle, involving seemingly minor, everyday instances of stereotyping that nonetheless accrue to produce significant negative effects on women (Valian, 1998). Although much of the research on subtle forms of gender bias focuses on its effects on women who report being the targets of bias, there has been less focus on the effects of merely witnessing bias directed at others. Moreover, we know little about the effects of witnessing subtle gender bias on people who are not typically the targets of bias, including men. The goal of the current research was to utilize an experimental approach to examine how both women and men are affected by witnessing subtle gender bias, in the form of *gendered microaggressions*, enacted toward women in small group interactions in a STEM context.

### 1.1. Gendered Microaggressions as Subtle Expressions of Gender Stereotypes

The term microaggressions refers to “brief, everyday exchanges that send denigrating messages to certain individuals because of their group membership” (Sue, 2010). In the current research, we consider microaggressions to be subtle behavioral expressions of stereotypes, or beliefs held about the characteristics, traits, and abilities of people based on their social group membership. Gender stereotypes reflect general beliefs that women are communal, warm, and nurturing, whereas men are agentic, competent, and logical (Eagly & Steffen, 1984). Following these general stereotypes, women are also stereotyped to have lower ability in mathematics and science compared to men (Bloodhart et al., 2020; Nosek et al., 2002b). Because our studies focus on small group interactions in the STEM context, we conceptualize gendered microaggressions as subtle behavioral expressions of gender-STEM stereotypes (e.g., the assumption that women are less competent than men in STEM) (see Table 1), focusing on instances in which men engage in gendered microaggressions directed at women.

We acknowledge that women can also hold gender-STEM stereotypes about other women (Nosek et al., 2002a), and can, therefore, engage in gendered microaggressions directed at other women, and men can be the targets of behaviors reflecting stereotypes about men. However, because our focus is on male-dominated STEM fields (i.e., computer science and engineering) and in such settings, women, not men, are negatively stereotyped regarding performance, we focus here on instances in which women are the targets of gendered microaggressions enacted by men in STEM settings.

### 1.2. Witnessing Gendered Microaggressions

Frequently being targeted by microaggressions is associated with a host of negative outcomes, such as depression, anxiety, lowered job satisfaction, and worse physical health (Nadal et al., 2013; Wong et al., 2014; Wright & Lewis, 2020). Relevant to the STEM context, prior research finds that women frequently report being the target of microaggressions in professional settings, often in the form of questioning or dismissing women’s competence and skills or expecting women to take on supportive and communal roles as opposed to technical and leadership roles (Hutchison, 2020; Yee et al., 2018). For example, in one study, 64% of women in a work setting reported being targeted by gendered microaggressions, largely related to presumed incompetence (Yee et al., 2018). Moreover, experiencing microaggressions predicts lowered motivation to pursue STEM education and employment, and diminished career advancement in STEM fields (Kim & Meister, 2023; Sekaquaptewa, 2019; Yang & Carroll, 2018).

While microaggression research has underscored the prevalence and consequences of experiencing gendered microaggressions, less research has focused on the consequences of being a mere witness to microaggressions. To the extent that women are often targeted by microaggressions in STEM settings, it is likely that many others are witnesses to gendered microaggressions. In support of this notion, we conducted a preliminary survey on Amazon Mechanical Turk (*N* = 201 STEM students) to assess the frequency of witnessing gendered microaggressions (specifically, behaviors implying that one presumes another is incompetent in STEM) directed at women compared to seeing these same behaviors directed at men or directed at oneself in STEM settings. Results indicated that both male and female students most often reported witnessing behaviors implying presumed incompetence in STEM directed at women, compared to presumed incompetence directed at men or directed at themselves (for a full reporting of the preliminary study, refer to the online Supplementary Materials, Section S1). The greater reporting of witnessing gendered microaggressions targeting women makes it important to examine the consequences of this on witnesses. Therefore, we focus on establishing the causal impacts of witnessing microaggressions targeting women, using an experimental approach.

### 1.3. Activating Social Identity Threat

Because gendered microaggressions communicate gender-STEM stereotypes, witnessing them can serve as a social identity threat cue for female students in male-dominated STEM fields. Social identity threat is a threat that occurs when people perceive they will be devalued or stereotyped in terms of one of their social identities (Steele et al., 2002). Individuals draw information from situational cues that convey messages about their group’s value and status in a setting. Exposure to social identity threat cues can be harmful to minoritized group members by negatively impacting their cognitions, affect, and behaviors related to the identity-threatening environment (Murphy et al., 2007). Examples of social identity threat cues for women in STEM include seeing very few other women present in a STEM context (Sekaquaptewa & Thompson, 2003) or being told about gender differences in math performance prior to taking a math test (Spencer et al., 1999). These settings send messages to women that they are devalued or negatively stereotyped in terms of their STEM competencies relative to men in such environments. Similarly, witnessing gendered microaggressions exposes people to behaviors that reflect gender-STEM stereotypes, and, thus, may serve as a social identity threat cue. For example, witnessing a gendered microaggression in the form of a woman being questioned about her presence in STEM (“You don’t look like an engineer!”) may bring to mind gender-STEM stereotypes that cast doubt on women’s STEM ability or competence, thereby negatively impacting one’s attitudes toward and performance in the stereotyped domain.

Experiencing a social identity threat has been shown to negatively impact women’s performance in STEM domains. In a classic example, when gender-math stereotypes become activated in a math testing situation (by telling women that gender differences have typically emerged on the test they were about to take, or simply stating that they were taking a math test), women showed diminished math test performance (Spencer et al., 1999). The activation of gender-STEM stereotypes has also been shown to diminish women’s test performance in math-intensive STEM fields, including engineering and physics (Logel et al., 2009; Miyake et al., 2010). Because social identity threat is theorized to diminish performance outcomes via psychological processes that reduce working memory capacity (Schmader & Johns, 2003), social identity threat cues, such as witnessing gendered microaggressions, may also diminish the recall of critical STEM information relayed in the situation (Taylor & Walton, 2011).

Beyond test performance, social identity threat cues also affect emotional and motivational outcomes, such as how enthusiastic women feel about participation in male-dominated settings (Cheryan et al., 2009; Dasgupta et al., 2015; Meadows & Sekaquaptewa, 2013; Murphy et al., 2007). Regarding microaggressions, student self-reports of witnessing racist and heterosexist microaggressions have been linked to lowered satisfaction with peer and faculty interactions (Amodeo et al., 2020; Watkins et al., 2010). Additionally, exposure to social identity threat cues has been found to activate concerns about appearing to confirm stereotypes about one’s group in the eyes of others, such as being seen as incompetent among women (Ramsey et al., 2013).

In the current research, we draw from the literature on social identity threats to examine the effects of witnessing gendered microaggressions in STEM group work contexts. Given research on social identity threat cues, we propose that exposure to stereotypes communicated by merely witnessing microaggressions targeting other women can be an identity-threatening cue to female witnesses that negatively impacts their recall of STEM information relayed in the group setting and enthusiasm for group work, and increases their concerns about being stereotyped by others as incompetent. These are important short-term outcomes because they not only diminish group performance (Emerson & Murphy, 2015; Peñalver et al., 2019) but may also lead to attrition in the long term. For instance, when a person working in a group is concerned about being negatively stereotyped by peers, fails to properly encode and recall critical information spoken by the group, or is unenthusiastic about working with the group, this could make it more likely that the person will disengage from the group setting.

### 1.4. Gender Differences in Effects of Witnessing Gendered Microaggressions

Because gender-STEM stereotypes implicate women as having a lower STEM ability than men, it seems likely that witnessing gendered microaggressions activates social identity concerns for women. But what about men? One study exposed a general sample of participants to videos of STEM faculty expressing gender bias toward women (e.g., favoring a less qualified male scientist over a woman) as well as men (e.g., discouraging a male graduate student from pursuing a teaching career), and found that such exposure to gender bias resulted in negative interpersonal outcomes, such as decreased affect and belonging, and increased concerns that their gender group will be stereotyped among women and among men (Pietri et al., 2019). However, it is unclear whether men would be impacted by witnessing gendered microaggressions targeting women even when men are not also the targets, as is the case in our conceptualization of gendered microaggressions. Moreover, in our studies, the participants were computer science and engineering students; thus, the men in our sample were chronically in a domain where they are positively stereotyped regarding their abilities. Because they are not the target of negative stereotypes about their ability in STEM, one could predict that men would not experience social identity concerns and negative group work outcomes associated with witnessing gendered microaggressions directed at women.

In our conceptualization of witnessing gendered microaggressions, men were the perpetrators of gender bias directed at a female target. Thus, male witnesses saw a gender in-group member—another man—as the stereotyper. This may raise concerns among men about stereotypes describing men as being disengaged from issues of sexism (Mahalik et al., 2012), or even about being perceived as sexist themselves. Indeed, research on interracial interactions suggests that privileged group members, specifically White Americans, do have identity-based concerns about appearing racially prejudiced to others (Frantz et al., 2004; Vorauer et al., 1998). When such identity-threatening cues are perceived (e.g., White participants are made to believe a discussion partner of another race sees them as prejudiced), White participants exhibit negative interpersonal outcomes, including negative affect and withdrawal from participating in interracial interactions (Bergsieker et al., 2010; Shelton et al., 2010; Vorauer & Turpie, 2004; Vorauer et al., 1998).

Similar to White participants’ concerns about being perceived as racist (Richeson & Shelton, 2007), witnessing men engage in gendered microaggressions in mixed-gender STEM settings could activate identity-based concerns about appearing sexist among male participants, and thus alter men’s experiences in the situation. For these reasons, it is possible that when witnessing gendered microaggressions, both men and women will become concerned that they will be judged in terms of stereotypes about their respective genders. Indeed, in our preliminary survey, among both male and female participants, witnessing a presumption of incompetence directed toward women was related to increased levels of gender stereotyping concerns (see the online Supplementary Materials, Section S1). Thus, among male witnesses, witnessing microaggressions may also result in stereotyping concerns, impaired recall of STEM information relayed in the group setting, and decreased enthusiasm for group work.

### 1.5. Stereotyping Concerns as a Mechanism

In addition to testing the effects of witnessing microaggressions on stereotyping concerns, recall, and enthusiasm among both female and male witnesses, in the current studies, we also examine whether the activation of stereotyping concerns accounts for decreases in enthusiasm and impaired recall. As described earlier, when stereotypes are made salient in a situation, people can become concerned about being stereotyped themselves (Steele, 2010). For women, witnessing microaggressions targeting women in a male-dominated STEM setting may activate concerns that their gender group could be presumed incompetent or lacking skills in STEM. While less is known about whether men (the dominant group) face stereotyping concerns, witnessing a man engage in microaggressions toward women may activate concerns that their gender group could be presumed sexist. The stereotype threat literature theorizes that the activation of stereotyping concerns can impact behaviors and attitudes (Steele et al., 2002; Steele, 2010). For instance, being concerned about how your group mates perceive you can reduce working memory capacity and impair encoding and recall of critical STEM information (Schmader & Johns, 2003). Additionally, being concerned about being stereotyped could result in lowered enthusiasm for group work. Therefore, the current studies examine whether the activation of stereotyping concerns from witnessing microaggressions partially accounts for a diminished recall of STEM information and enthusiasm about working with the group.

### 1.6. Overview of the Studies and Hypotheses

To investigate the effects of witnessing gendered microaggressions on women and men in STEM, we conducted three experiments in which computer science and engineering students watched a video of a four-person mixed-gender group of students working on an engineering task. The participants were randomly assigned to watch scenarios in which microaggressions occur in the project teams or neutral interactions occur. The first study was conducted in-person, in a laboratory setting in which students anticipated they would soon interact with the same group of peers to work on an engineering task, and the remaining two were conducted online and, thus, the participants did not anticipate interacting with a group (but were asked to imagine the prospect of working with the group depicted in the videos). This provided the opportunity to examine outcomes in situations in which participants anticipated actual interaction with a group and in which they did not. Our second and third studies are presented together as Studies 2a and 2b because one is a replication of the other. We focus on small group work contexts in STEM as much of student life takes place in the presence of peers; moreover, STEM courses, in particular, have increasingly used team-based pedagogical strategies because group work is beneficial to learning outcomes (Smith, 1996; Freeman et al., 2007; Haidet et al., 2014; Love et al., 2014; Severiens & Schmidt, 2009).

Because we define microaggressions as behavioral expressions of gender stereotypes that could serve as a social identity threat cue for female and male witnesses, in all the studies, we examined whether witnessing microaggressions would diminish the recall of STEM information spoken by the group and reduce enthusiasm about working with the group depicted in the video. We also examined whether witnessing microaggressions would activate gender stereotyping concerns among both men and women. Moreover, we tested whether the activation of stereotyping concerns from witnessing microaggressions accounts for diminished recall and enthusiasm. In Study 1, we measured gender stereotyping concerns in a general sense—that is, asking participants if they are concerned whether their performance in the group task will reflect on their gender group (i.e., not specifying the specific stereotype participants are concerned about confirming). In Studies 2a and 2b, we examined gender-specific stereotyping concerns, that is, the concern that one’s peers will presume one’s gender group to be incompetent (a stereotype more relevant to women) and sexist (a stereotype more relevant to men). Thus, we hypothesized the following:

**H1.**  *Witnessing microaggressions, compared to neutral interactions, in a STEM group work context, will diminish the recall of STEM information spoken by the group.*

**H2.**  *Witnessing microaggressions, compared to neutral interactions, in a STEM group work context, will reduce enthusiasm about working with the group.*

**H3.**  *Witnessing microaggressions, compared to neutral interactions, in a STEM group work context, will activate general stereotyping concerns (tested in Study 1). Female witnesses will be concerned that their gender group will be presumed incompetent among their peers, whereas male witnesses will be concerned that their gender group will be presumed sexist among their peers (tested in Studies 2a and 2b).*

**H4.**  *The activation of general stereotyping concerns from witnessing microaggressions (compared to neutral interactions) will partially account for diminished recall and enthusiasm (tested in Study 1). For female witnesses, concerns that their gender group is presumed incompetent will partially account for the relationship between witnessing microaggressions and diminished recall and enthusiasm. For male witnesses, concerns that their gender group is presumed sexist will partially account for the relationship between witnessing microaggressions and diminished recall and enthusiasm (tested in Studies 2a and 2b).*

## 2. Study 1

### 2.1. Method

#### 2.1.1. Participants

Undergraduates majoring in engineering or computer science were recruited for this study from a large midwestern US university (*N* = 226). Eligible students were identified by the university registrar’s office and were emailed recruitment announcements; students were also recruited through posted flyers on public bulletin boards. Students were monetarily compensated for their participation.

Eighteen participants (ten in the witnessing microaggressions condition, eight in the control condition) stated they did not believe the cover story during debriefing and were excluded from the sample. Because gender was a factor in the analyses, six participants who identified with a gender that was not man or woman were also excluded. This resulted in a final sample of 204 participants, of which 98 were men and 106 were women.^1^ The mean age of our sample was 19.57 years (*SD* = 1.60); 51% of the participants identified as White, 36% as Asian/Asian American, 2% as Black/African American, <1% as Latino/Hispanic, 10% selected more than one race/ethnicity, and <1% did not select a racial/ethnic category (note that due to a rounding error, the numbers do not add up to 100%).

The sample size was determined by the number of participants we were able to recruit within an academic year and our attempt to maintain a relatively gender-balanced sample (which required over-recruiting women). A post hoc power analysis determined we were 80% powered to detect an interaction effect between small- and medium-sized *f*^2^ = 0.039 (Champely, 2020; Cohen, 1988) and to detect a medium-sized indirect effect for each man and woman of around *B* = 0.15 (Fritz & MacKinnon, 2007).

#### 2.1.2. Procedure and Materials

The participants attended a 60 min in-lab session. The lab space included individual private cubicles as well as a main room set up to resemble a workspace for a team project (i.e., chairs around a table equipped with scrap paper, pencils, and calculators, and a whiteboard in the room). The participants reported to the lab individually or with one other participant. They completed the informed consent form in the main room, then were seated in a private cubicle and completed the study on the computer in the cubicle.

The participants were told a cover story that the purpose of the study was to examine the experience of someone who joins a group engineering project as the new team member in an already existing group. The participants believed they would complete some individual tasks and then join an already existing group of students to work on an engineering project.^2^ As part of the cover story, the participants were told that some participants in the study were “randomly assigned” to join the group with no prior knowledge of the group, whereas others would gain some prior knowledge of the group beforehand by watching a short compilation of video clips of the team working together in a previous session. All participants were told they had been assigned to the “prior knowledge” condition and watched one of two videos showing the group they believed they would engage with on an engineering task.

The videos constituted the experimental manipulation. Two video versions were developed, each showing a group of four college students working on an ocean engineering project to design a floating buoy that measures nearshore water flow to acquire data on rip currents that may increase safety for swimmers. The participants anticipated working on this same task with the group. In both video versions, the group of college students is shown seated around a table discussing their group project, such as assigning tasks and brainstorming ideas (e.g., how to launch their buoy from shore), as well as engaging in unrelated side conversations (e.g., talking about other classes). The four student actors displayed in the videos were two men and two women. Regarding the racial makeup of the video actors, both men were White, one woman was White, and one woman was Asian/Asian American. The actors were the same in both video versions.

The video compilations each showed nine clips of similar length (totaling ~5 min). In the microaggression condition, five clips showed gendered microaggressions occurring in the students’ interactions, and the remaining four showed neutral interactions. In the control condition, all clips showed neutral interactions (see Table 1). In both video versions, the content the students discussed was the same; however, the microaggression version displayed interactions wherein the male students were engaging in microaggression behaviors toward the female students. (e.g., in clip 3, in both the microaggression and control versions, students discuss what color the buoy should be. In the control version, a female student suggests orange would be a visible color, and her idea is discussed and accepted. In the microaggression version, the female student also suggests orange, but her idea is ignored, and then later, a man repeats the same idea and is given credit for it).

**Table 1:** Descriptions of microaggressions and control video versions.

Clip Number	Microaggression Version		Control Version
	Stereotypic Interaction	Stereotype Reflected	Non-Stereotypic Interaction
1	A man asks a woman to take the secretarial role of note-taker.	Women primarily support men’s work in STEM and adopt stereotypic roles such as secretary.	A man volunteers to take notes.
2	Students discuss being in research studies.	None; neutral interaction	Students discuss being in research studies.
3	A woman’s idea is ignored until a man repeats it and is given credit for it.	Men are more credible sources of good ideas in STEM than women.	A woman’s idea is discussed and accepted.
4	Students discuss their summer vacations.	None; neutral interaction	Students discuss their summer vacations.
5	A woman volunteers ideas but the men speak over her.	Women’s STEM contributions are not as important as men’s.	A woman volunteers ideas without being spoken over.
6	A man expresses surprise that a woman is in a more advanced calculus class than he is in.	It is unusual and unexpected for women to be highly competent in STEM.	A woman states she is in an advanced calculus class without comment from others.
7	Students discuss their internet research.	None; neutral interaction	Students discuss their internet research.
8	A man explains a concept to a woman after she states that she is already familiar with the concept.	Women are assumed to be less knowledgeable than men.	A man and a woman discuss a concept that they are both familiar with.
9	A woman reads the project instructions and requirements to the group.	None; neutral interaction	A woman reads the project instructions and requirements to the group.

Note. Clips 2, 4, 7, and 9 were identical in both video versions. Clip 9 provides the engineering-related material that participants were later asked to remember. To see the full video transcripts, along with all the survey items that constitute the measures reported in the main text for each study, see the online Supplementary Materials, Section S9.

Video scripts were pretested to ensure that the microaggression video version was perceived as depicting significantly more gender-STEM stereotyping than the control version (pretesting details are presented in the online Supplementary Materials, Section S2).^3^

After watching the video, the participants completed a questionnaire on the computer. After the participants completed these measures, the experimenter returned to inform them that they would not be joining the group for the group task after all. The participants then engaged in an extended debriefing to assess the participants’ suspicion of the cover story as well as to inform them of the true nature and goals of the study.

### 2.2. Measures

**Recall of engineering information discussed by the group.** The participants answered five items about the engineering-relevant information discussed by the group in the video they watched earlier. Each item consisted of four multiple-choice response options, and their recall of the engineering information was measured by the number of correct answers. An example item is as follows: What is the purpose of the buoy being designed? A. to measure rip currents, B. to measure flow in a river, C. to measure boat speed, D. to mark a swimming area.

**Enthusiasm about working with the group.** Three items were averaged together to assess enthusiasm about working with the group (e.g., “I look forward to working with this group”, 1 = Strongly disagree to 7 = Strongly agree). Higher scores indicated greater enthusiasm (α = 0.86).

**General stereotyping concerns.** Participants were asked two items to assess gender stereotyping concerns (e.g., “I am concerned that others will judge people of my gender as a whole based on my performance in the group task”, 1 = Strongly disagree to 7 = Strongly agree), adapted from Ramsey et al. (2013). Higher scores indicated greater gender stereotyping concerns (*r* = 0.84).^4^

### 2.3. Results

#### 2.3.1. Two-Way ANOVAs

The study used a 2(condition: microaggression vs. control) × 2(participant gender: man vs. woman) between-subjects design. For each outcome variable, a two-way ANOVA was used to assess the effect of the condition and the interaction with gender. Only significant effects will be elaborated on in the main text. The full ANOVA results for each outcome can be found in the online Supplementary Materials, Section S4. Correlations between all the dependent variables for all the studies can be found in the online Supplementary Materials, Section S6.

**Recall of engineering information discussed by the group.** A significant main effect of the condition emerged, (*F*(1, 199) = 9.31, *p* = 0.003), such that the participants in the control condition (*M* = 3.73, *SD* = 1.09) had a better memory of the engineering information than those in the microaggression condition (*M* = 3.22, *SD* = 1.23). No significant main effects of gender or interaction emerged; all *F*s < 2.60, all *p*s > 0.10.

**Enthusiasm about working with the group.** The analyses revealed a significant main effect of the condition, *F*(1, 200) = 40.04, *p* < 0.001, such that the participants in the control condition (*M* = 4.76, *SD* = 1.00) had greater enthusiasm about working with the group than did those in the microaggression condition (*M* = 3.82, *SD* = 1.14). No significant main effects of gender or interaction emerged; all *F*s < 1.40, all *p*s > 0.24.

**General stereotyping concerns.** There was a significant main effect of gender, *F*(1, 200) = 128.05, *p* < 0.001, such that women (*M* = 3.99, *SD* = 1.64) reported more gender stereotyping concerns than men (*M* = 1.84, *SD* = 1.01). A significant main effect of the condition also emerged, *F*(1, 200) = 13.09, *p* < 0.001, such that the participants in the control condition (*M* = 2.57, *SD* = 1.55) reported less concern that others would stereotype them in terms of gender compared to participants who witnessed microaggressions (*M* = 3.36, *SD* = 1.84). No significant interaction emerged; *F*(1, 200) = 1.39, *p* = 0.241.

#### 2.3.2. Multigroup Path Analysis

We hypothesized that stereotyping concerns would partially explain the relationship between witnessing microaggressions and a decreased recall of engineering information as well as enthusiasm for working with the group. To examine whether stereotyping concerns partially account for the effect of this condition on the recall of engineering information for both female and male students, we used a multigroup path analysis approach, with participant gender as our grouping variable. Path analyses were conducted in R-Studio (Version 2023.06) using the Lavaan package, and 10,000 bootstraps were conducted to estimate 95% confidence intervals around the indirect effect (Rosseel, 2012). Even though there was not a significant condition-by-gender interaction effect on our recall or enthusiasm outcomes, we still included gender as a grouping variable for our path analysis models to examine whether the relationship between stereotyping concerns and our outcomes, as well as the indirect effect, differed for male and female students.

**Figure 1:**
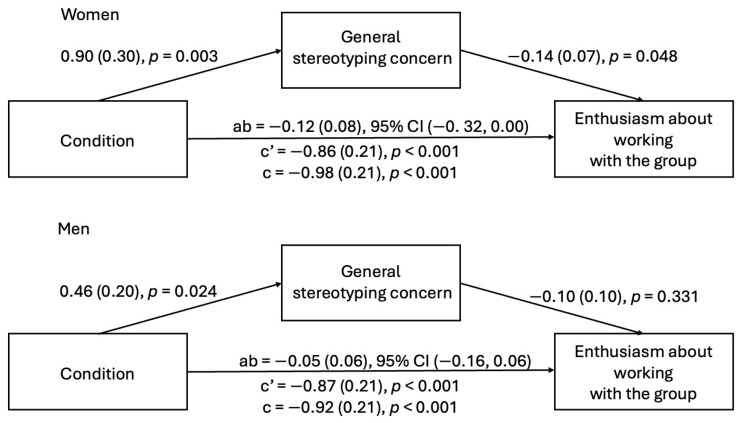
Indirect effect of condition on enthusiasm for group work through general stereotyping concerns (Study 1).

**Enthusiasm about working with the group.** First, we tested whether stereotyping concerns partially accounted for the relationship between witnessing microaggressions and decreased enthusiasm among women and men. For both women and men, witnessing microaggressions heightened stereotyping concerns. For women, but not men, stereotyping concerns were also related to lowered enthusiasm for group work. However, the indirect effect was not statistically significant for women or men, although for women, both the *a* and *b* paths in the model were significant (see Figure 1).

**Figure 2:**
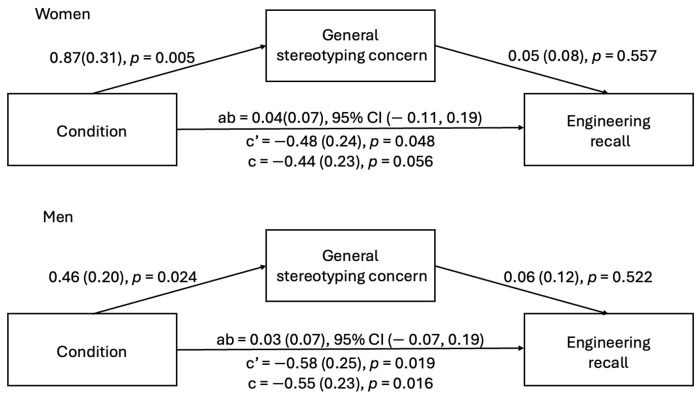
Indirect effect of condition on engineering recall through general stereotyping concerns (Study 1).

**Recall of engineering information discussed by the group.** Next, we tested whether stereotyping concerns partially account for the relationship between witnessing microaggressions and the decreased recall of engineering information among women and men. For both women and men, while witnessing microaggressions predicted increased stereotyping concerns, stereotyping concern did not relate to decreased engineering recall. Thus, the indirect effect was not significant for men or women (see Figure 2).

### 2.4. Study 1 Discussion

Among both men and women, witnessing microaggressions, compared to neutral interactions, resulted in a diminished recall of the engineering information discussed by the group in the video, less enthusiasm about working with the group depicted in the video, and greater concerns about being stereotyped by the group according to one’s gender (confirming Hypotheses 1–3). This suggests that, for both women and men, learning and working in STEM settings where some peers engage in microaggressions may lead to more negative feelings about collaborating with their peers and increased concern about being stereotyped by them. The presence of microaggressions may also distract from learning critical information for both female and male students. In Study 1, inconsistent with Hypothesis 4, we did not find evidence to suggest that the activation of stereotyping concerns partially accounts for diminished enthusiasm and recall due to witnessing microaggressions. Although, for women, the indirect effect did not reach statistical significance (i.e., the confidence interval includes 0), both the a and b paths were consistent with predictions, such that witnessing microaggressions increased stereotyping concerns and stereotyping concerns related to lowered enthusiasm.

Overall, the results from Study 1 are consistent with the self-report literature suggesting negative academic and interpersonal consequences associated with experiencing microaggressions in STEM (Kim & Meister, 2023; Yang & Carroll, 2018). Our results are concerning as much academic learning takes place in the context of group work, and witnessing microaggressions may interfere with the learning benefits derived from group work, as well as its interpersonal benefits, such as forming social connections (Freeman et al., 2007; Haidet et al., 2014; Love et al., 2014). Moreover, witnessing microaggressions may distract people from properly encoding and then later recalling STEM-relevant information relayed by their peers, which could significantly impair learning and later performance.

## 3. Studies 2a and 2b

In Studies 2a and 2b, we replicated and extended the findings from Study 1. Studies 2a and 2b were conducted online due to the COVID-19 pandemic. One of the primary goals of Studies 2a and 2b was to more directly examine the specific type of stereotyping concerns experienced by men and women after witnessing microaggressions. As women tend to be stereotyped as incompetent in STEM and men may be stereotyped as being sexist (Bloodhart et al., 2020; Mahalik et al., 2012; Nosek et al., 2002b), we predicted that for women, witnessing gendered microaggressions would primarily elicit stereotyping concerns regarding their presumed incompetence, whereas for men, it would primarily elicit concerns regarding their presumed sexism.^5^ Additionally, in Studies 2a and 2b, we assess whether the activation of these gender-specific stereotyping concerns partially explains the relationship between witnessing microaggressions and impaired memory of engineering information and lowered enthusiasm for group work.

### 3.1. Method

#### 3.1.1. Participants

**Study 2a.** Participants were recruited via Prolific (an online research recruitment platform), and with its pre-screeners, we recruited 412 college students majoring in engineering or computer science. The participants were monetarily compensated for their participation. We excluded people from this sample who failed either of the two attention checks we included in the study, self-indicated that their data were not of good quality, did not identify as current college students, or reported they were not majoring in engineering or computer science in our internal survey. This left us with a final sample of 306 participants. Table 2 provides a summary of the exclusion criteria and participant demographic characteristics in this study. A post hoc power analysis determined we were 80% powered to detect an interaction effect between small- and medium-sized *f*^2^ = 0.026 (Champely, 2020; Cohen, 1988) and to detect a small-to-medium indirect effect for each man and woman of around *B* = 0.10 (Fritz & MacKinnon, 2007).

**Study 2b.** In Study 2b, we recruited 272 US college students majoring in engineering or computer science, again from Prolific (excluding participants who had participated in Study 2a).^6^ The participants were monetarily compensated for their participation. We excluded people from this sample who failed either of the two attention checks we included in the study, who did not identify as men or women in our internal survey, or who did not identify as current US residents for at least 5 years. This left us with a final sample of 243 participants. A summary of exclusion criteria and participant demographic characteristics is provided in Table 2.

Our goal was to have a final sample of 300 participants (similar to Study 2a, and a gender-balanced sample); this was determined by an a priori power analysis indicating we would be powered to detect interaction effects of *f*^2^ = 0.027 (Champely, 2020; Cohen, 1988) and a small/medium indirect effect for each man and woman of *B* = 0.07 (note, this effect size is slightly smaller than that of Study 2a due to a gender imbalance in 2a) (Fritz & MacKinnon, 2007). However, this was determined to not be feasible as it appeared we had exhausted the pool of potential female respondents (i.e., there was single-digit participation per day after leaving data collection open for two weeks with multiple study postings). With our final sample, a post hoc power analysis indicated we were powered to detect an interaction effect of *f*^2^ = 0.033 and to detect a small-to-medium indirect effect for each man and woman of around *B* = 0.10 (Fritz & MacKinnon, 2007).

#### 3.1.2. Procedure

In both Studies 2a and 2b, participants who qualified based on the pre-screening criteria were invited to take part in an online study described as examining people’s thoughts and feelings about a team’s interactions. Participants were told that we had filmed clips of team interactions from multiple groups of engineering students working on a group project and that they would be randomly assigned to watch clips from one of these teams. Because the study was online, participants knew they would not actually be interacting with the team later; instead, they were asked to imagine that they would shortly join the team they saw and report their thoughts and feelings about that prospect. The video manipulation was the same as in Study 1. Participants were randomly assigned to watch the video depicting neutral team interactions (control condition) or men enacting microaggressions against women (microaggression condition). After watching the video, participants proceeded to the outcome measures and were then debriefed.

#### 3.1.3. Measures

**Engineering information recall.** In both Studies 2a and 2b, participants answered the same 5-item questionnaire about the engineering-relevant information presented, and scoring was conducted the same way as in Study 1 (i.e., total correct sum score).

**Enthusiasm about working with the group.** In both Studies 2a and 2b, participants answered the same 3-item questionnaire about enthusiasm about working with the group as they did in Study 1; however, the items were worded to reflect the online nature of Studies 2a and 2b (e.g., “I would look forward to working with this group”, 1 = Strongly disagree to 7 = Strongly agree) (Study 2a α = 0.92; Study 2b α = 0.94).

**Assumed sexist stereotyping concerns.** In both Studies 2a and 2b, concerns about appearing sexist were assessed with two items (e.g., “I am concerned that other people in the group will assume people of my gender are sexist”, 1 = Strongly disagree to 7 = Strongly agree) (Study 2a *r* = 0.76; Study 2b *r* = 0.66). Higher numbers indicate higher levels of concern that others would see your gender as biased toward the other gender.

**Assumed incompetent stereotyping concerns.** In both Studies 2a and 2b, concerns about one’s gender being stereotyped as incompetent in computer science and engineering were assessed with a three item questionnaire (e.g., “I am concerned that other people in the group will assume people of my gender are unskilled in science and engineering”, 1 = Strongly disagree to 7 = Strongly agree) (Study 2a α = 0.96; Study 2b α = 0.97).

### 3.2. Results

#### 3.2.1. Two-Way ANOVAs

For each of the engineering recall and enthusiasm for group work outcomes 2(condition: microaggression vs. control) × 2(participant gender: man vs. woman) between-subjects ANOVAs were conducted. The full ANOVA results can be found in the online Supplementary Materials, Section S4; only the significant effects will be elaborated on in the main text.

**Engineering information recall.** Inconsistent with Study 1, in both Studies 2a and 2b, no significant main effects or interactions emerged for recall of engineering information; all *F*s < 1.15, all *p*s > 0.33.

**Enthusiasm about working with the group.**

***Study 2a***. Analyses revealed a significant main effect of the condition, *F*(1, 302) = 92.85, *p* < 0.001, such that participants in the control condition (*M* = 4.87, *SD* = 1.24) had more enthusiasm about working with the group than did those in the microaggression condition (*M* = 3.46, *SD* = 1.37). No other significant main effects or interactions of interest emerged (all *F*s < 3.45, all *p*s > 0.05); however, the condition-by-gender interaction effect was marginally significant (*p* = 0.063), such that the effect of witnessing microaggressions on diminished enthusiasm was larger for female than male witnesses.

**Figure 3:**
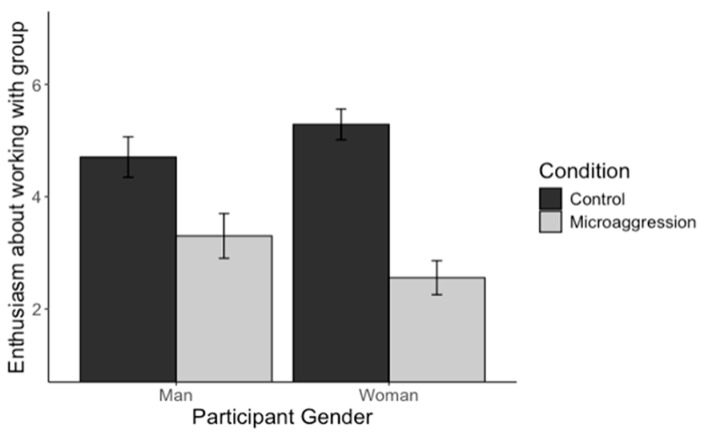
Mean and 95% confidence interval of the mean for enthusiasm by participant gender and condition (Study 2b).

***Study 2b.*** Analyses revealed a significant main effect of the condition, *F*(1, 239) = 148.10, *p* < 0.001, and a non-significant effect of gender, *F*(1, 239) = 0.23, *p* = 0.631. These were qualified by a significant two-way interaction, *F*(1, 239), 15.22, *p* < 0.001. Simple main effects analysis revealed that witnessing microaggressions compared to neutral interactions decreased enthusiasm among male, *F*(1, 120) = 26.89, *p* < 0.001, and female participants, *F*(1, 119) = 178.76, *p* < 0.001; however, the effect of the condition was larger for women (see Figure 3).

#### 3.2.2. Mixed ANOVA—Gender Stereotyping Concerns

To assess what specific aspects of gender stereotyping male and female participants were concerned about in response to witnessing microaggressions, in both Studies 2a and 2b, we conducted a 2(condition: microaggression vs. control) × 2(gender: men vs. women) × 2(stereotyping concern type: incompetent vs. sexist) mixed ANOVA. See Table 3 for the full reporting of ANOVA results. Significant three-way interactions were decomposed into simple two-way interactions and then simple main effects (Bruin, 2006; Kassambara, 2018).

**Figure 4:**
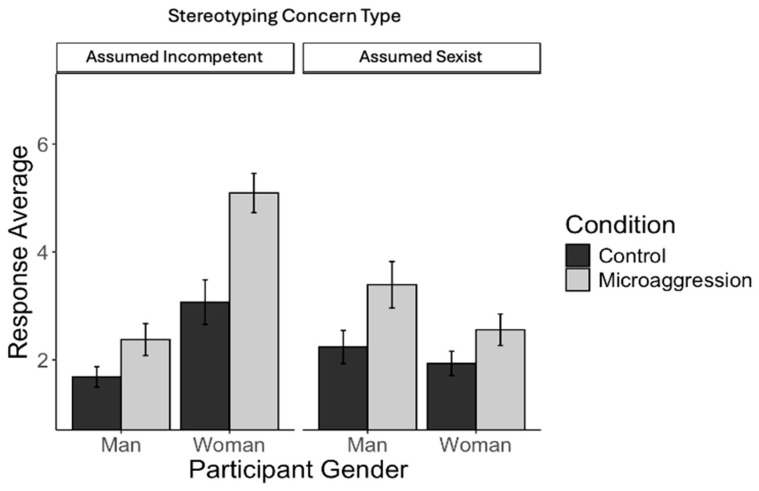
Mean and 95% confidence interval of the mean for stereotyping concerns by participant gender, condition, and concern type (Study 2a).

**Study 2a.** For Study 2a, the mixed ANOVA revealed a significant three-way interaction; see Figure 4 for a graph of means and the 95% confidence interval of the mean for the three-way interaction. We decomposed the interaction into two simple condition × gender interactions for assumed incompetent and assumed sexist stereotyping concerns.

For assumed sexist concerns, there was no significant condition × gender interaction; *F*(1, 302) = 2.36, *p* = 0.125. However, there was a main effect of gender, *F*(1, 302) = 10.86, *p* = 0.001, such that men expressed higher concerns about being presumed sexist compared to women. There was also a main effect of the condition, *F*(1, 302) = 26.50, *p* < 0.001, such that participants were more concerned about being presumed sexist in the microaggression compared to the control condition.

For assumed incompetent stereotyping concerns, there was a significant effect of that condition, *F*(1, 302) = 76.68, *p* < 0.001, and gender, *F*(1, 302) = 174.88, *p* < 0.001, which were qualified by a significant two-way interaction, *F*(1, 302) = 18.42, *p* < 0.001. This two-way interaction was followed up with a simple main effect test to examine the effect of the condition on assumed incompetent stereotyping concerns among men and women. This revealed that witnessing microaggressions compared to neutral interactions increased stereotyping concerns about presumed incompetence among men, *F*(1, 174) = 16.01, *p* < 0.001, and women, *F*(1, 128) = 54.27, *p* < 0.001; however, the effect was greater among women.

**Figure 5:**
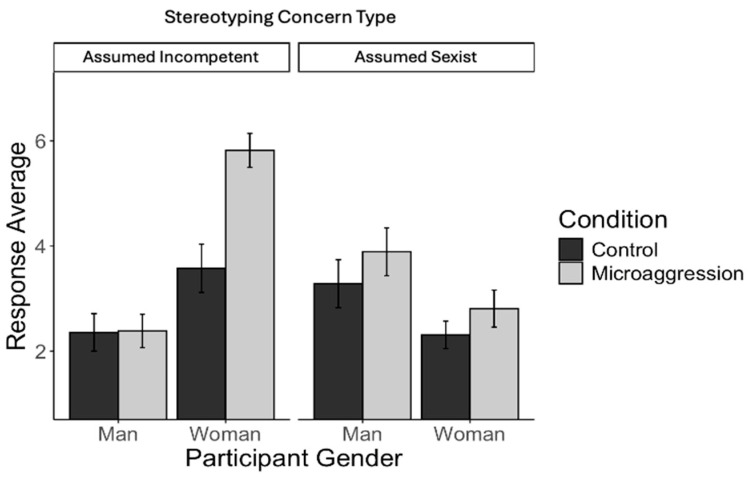
Mean and 95% confidence interval of the mean for stereotyping concerns by participant gender, condition, and concern type (Study 2b).

**Study 2b.** Study 2b replicated Study 2a as the mixed ANOVA revealed a significant three-way interaction, *F*(1, 239) = 29.24, *p* < 0.001; see Figure 5 for a graph of means and the 95% confidence interval of the mean for the three-way interaction. We decomposed the interaction into two simple condition × gender interactions for presumed incompetent and presumed sexist stereotyping concerns.

For presumed sexist stereotyping concerns, there was no significant condition × gender interaction; *F*(1, 239) = 0.08, *p* = 0.782. However, there was a main effect of gender, *F*(1, 239) = 28.02, *p* < 0.001, such that men expressed higher concerns about being presumed sexist compared to women. There was also a main effect of the condition, *F*(1, 239) = 8.09, *p* = 0.005, such that participants were more concerned about being presumed sexist in the microaggression compared to the control condition.

For assumed incompetence stereotyping concerns, there was a significant effect of that condition, *F*(1, 239) = 36.49, *p* < 0.001, and gender, *F*(1, 239) = 153.05, *p* < 0.001, which were qualified by a significant two-way interaction, *F*(1, 239) = 34.64, *p* < 0.001. This two-way interaction was followed up with a simple main effect test to examine the effect of the condition on assumed incompetence stereotyping concerns among men and women. This revealed that witnessing microaggressions compared to neutral interactions increased stereotyping concerns about being presumed incompetent among women, *F*(1, 119) = 59.17, *p* < 0.001, but not among men, *F*(1, 120) = 0.02, *p* = 0.903.

Thus, taken together, the mixed ANOVA results from Studies 2a and 2b indicate that for female participants, witnessing microaggressions activates concerns about being presumed incompetent. While male participants were, overall, more concerned about appearing sexist than female participants, witnessing microaggressions seemed to elevate stereotyping concerns of either type less for men than women (see Figure 4 and Figure 5).

#### 3.2.3. Multigroup Path Analysis

We hypothesized that for women, concerns that one’s gender would be presumed incompetent would partially explain the relationship between witnessing microaggressions and decreased enthusiasm for working with the group, whereas for men, concerns that one’s gender would be presumed sexist would partially explain this relationship. We used a multigroup path analysis to test these models, with participant gender as our grouping variable. Path analyses were conducted in R using the Lavaan package (Rosseel, 2012), and 10,000 bootstraps were conducted to estimate 95% confidence intervals around the indirect effect.

Although, a priori, we hypothesized that the activation of stereotyping concerns from witnessing microaggressions could also partially account for impaired recall, we did not test mediation models with the multiple-choice engineering recall outcomes as there was not a significant effect of that condition on this outcome for either Study 2a or 2b.

**Mediator—assumed sexist stereotyping concern.** First, we tested whether concerns about being presumed sexist partially account for the relationship between witnessing microaggressions and decreased enthusiasm among women and men.

**Figure 6:**
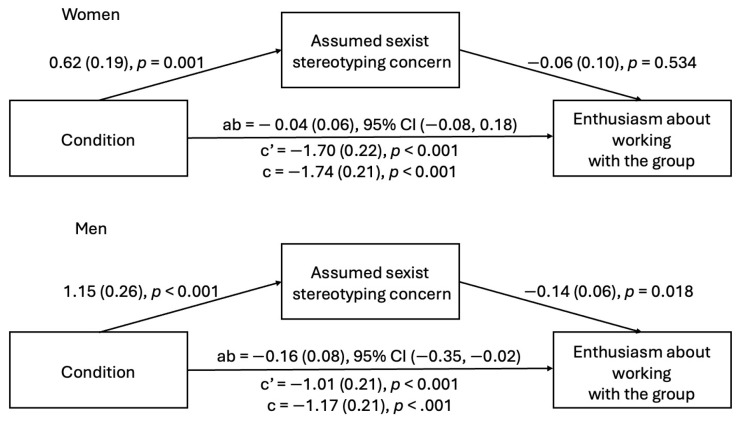
Indirect effect of condition on enthusiasm through assumed sexist stereotyping concern (Study 2a).

***Study 2a.*** In Study 2a, witnessing microaggressions predicted increased gender stereotyping concerns about being presumed sexist among both men and women. However, for men but not women, the presence of gender stereotyping concerns about being presumed sexist was related to decreased enthusiasm about working with the group. Additionally, the indirect effect was significant for men but not women (see Figure 6). These results indicated that concern about being presumed sexist partially explained the relationship between witnessing microaggressions and decreased enthusiasm about working with the group among male participants.

**Figure 7:**
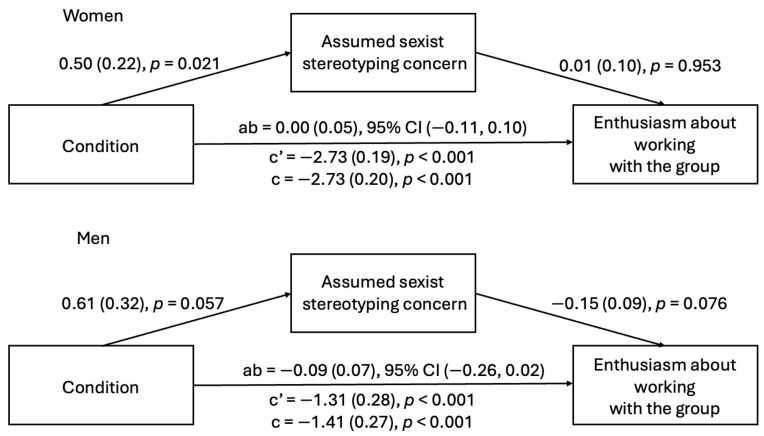
Indirect effect of condition on enthusiasm through assumed sexist stereotyping concern (Study 2b).

***Study 2b.*** In Study 2b, witnessing microaggressions predicted increased gender stereotyping concern about being presumed sexist among women, but this relationship was only marginally significant among men. For both women and men, gender stereotyping concern about being presumed sexist was not related to decreased enthusiasm about working with the group. Thus, the indirect effect was not significant for either women or men, in contrast to the Study 2a findings (however, it was trending in the same direction as in Study 2a for men) (see Figure 7).

**Mediator—assumed incompetent stereotyping concern.** Next, we tested whether concerns about being presumed incompetent partially account for the relationship between witnessing microaggressions and decreased enthusiasm among women and men.

**Figure 8:**
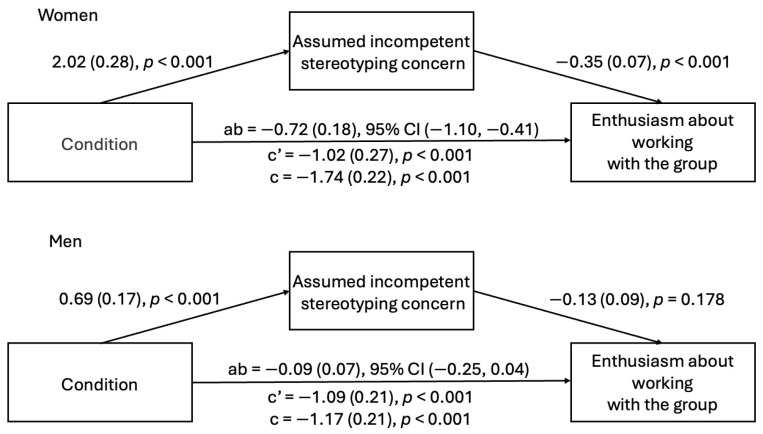
Indirect effect of condition on enthusiasm through assumed incompetent stereotyping concern (Study 2a).

***Study 2a.*** Witnessing microaggressions predicted increased gender stereotyping concern about being presumed incompetent among both men and women. For women, but not men, concern about being presumed incompetent was related to decreased enthusiasm about working with the group. Additionally, the indirect effect was significant for women but not men (see Figure 8). These results indicated that gender stereotyping concern about being presumed incompetent partially explained the relationship between witnessing microaggressions and decreased enthusiasm about working with the group among female participants.

**Figure 9:**
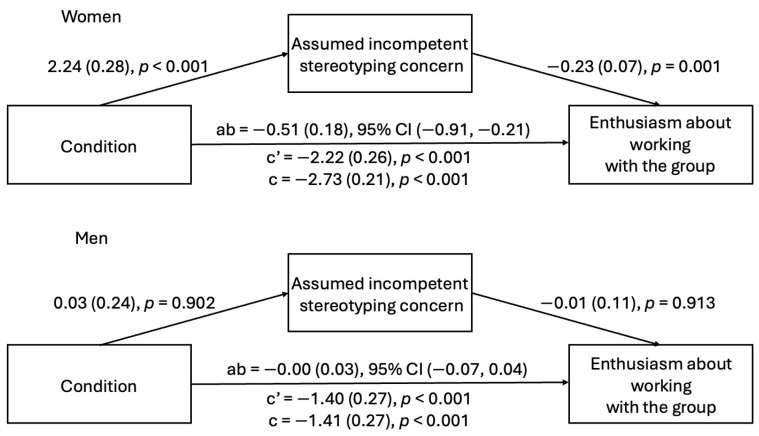
Indirect effect of condition on enthusiasm through assumed incompetent stereotyping concern (Study 2b).

***Study 2b.*** In Study 2b, witnessing microaggressions predicted increased gender stereotyping concern about being presumed incompetent among women, but not among men. Similarly, for women, but not for men, gender stereotyping concern about being presumed incompetent was related to decreased enthusiasm about working with the group. Thus, the indirect effect was only significant for women (see Figure 9). These results indicated that gender stereotyping concern about being presumed incompetent partially explained the relationship between witnessing microaggressions and decreased enthusiasm about working with the group among female participants, replicating Study 2a.

### 3.3. Study 2a and Study 2b Discussion

Replicating findings from Study 1, both men and women showed less enthusiasm about working with a group after viewing gendered microaggressions occurring in that group in Studies 2a and 2b (again, confirming Hypothesis 2). Additionally, in Study 2b, the effect of the condition on enthusiasm was greater for female than male participants (in Study 2a, this interaction was trending in the same direction but was not statistically significant).

Contrary to Hypothesis 1, in both Studies 2a and 2b, there was not a significant effect of witnessing microaggressions on recall of the engineering information discussed by the group. This is in contrast to Study 1, wherein witnessing microaggressions—compared to the control condition—resulted in lower scores on the same multiple-choice test. This discrepancy may be a result of the different experiences across the studies. As Study 1 was in person, the participants believed they would soon work with the same engineering team on a similar task, whereas in Studies 2a and 2b (due to the online format), the participants were simply told to imagine the prospect of working with the group depicted in the videos. Thus, with the in-person setting in Study 1, the witnessed microaggressions may have felt more personally consequential (due to concerns about soon working with the observed group) and more strongly distracted participants from the engineering information presented in the videos, compared to Studies 2a and 2b. This possibility is aligned with research indicating stronger effects of manipulations in real interactions compared to imagined ones (Clifford & Jerit, 2014; Finley & Penningroth, 2015).

Studies 2a and 2b also extended the general gender stereotyping concerns outcome in Study 1 by demonstrating that gender-specific stereotyping concerns differed among male and female witnesses (confirming Hypothesis 3).^7^ Across both Studies 2a and 2b, women were more concerned than men about being presumed incompetent after witnessing microaggressions, and men were more concerned than women about appearing sexist (indeed, an additive effect of that condition and gender emerged for appearing sexist concerns, rather than an interaction). Importantly, for women—not men—concerns about being presumed incompetent partially accounted for the effects of witnessing gendered microaggressions on decreased enthusiasm for group work (partially confirming Hypothesis 4). There was some evidence, although inconsistent, that for men, stereotyping concerns about appearing sexist partially explained the relationship between witnessing microaggressions and decreased enthusiasm. These results suggest that for women, in particular, witnessing microaggressions against another woman raises concerns about one’s gender group being presumed incompetent, which can inhibit positive group work experiences.

## 4. General Discussion

In three experiments, we tested the hypothesis that witnessing gendered microaggressions (subtle behavioral expressions of gender-STEM stereotypes) would diminish outcomes for both men and women in a STEM context. Men and women STEM undergraduates were randomly assigned to witness either a series of gendered microaggressions perpetrated by men toward women in a group interaction or a series of neutral interactions in an in-person experiment (Study 1) and in virtual (online) experiments (Studies 2a and 2b).

The results showed that witnessing gendered microaggressions reduced enthusiasm for group work among both men and women, an effect that was significantly stronger among women in one study. This finding is important given the growing trends in STEM education toward learning through collaborative group projects and working in teams. Learning STEM principles through small group work has been shown to produce positive outcomes, including higher achievement and greater productivity, positive relationships, and increased self-esteem (Smith, 1996). Our results suggest that the benefits of group work are diminished when students see their peers engage in subtle behaviors communicating negative stereotypes about women in STEM. Given stereotypes disfavoring women’s competence and skill in STEM (Nosek et al., 2002b), witnessing gendered microaggressions among one’s peers may be a common experience for STEM students.

Witnessing gendered microaggressions also increased gender stereotyping concerns among both men and women. These concerns were assessed both generally (not specifying the stereotype participants are concerned about confirming) and in terms of gender stereotypes specific to men and women, i.e., the stereotype that women are incompetent in STEM and the stereotype that men are sexist. This suggests that witnessing gendered microaggressions raises concerns about stereotyping among not only women but men as well, with women becoming concerned about being presumed incompetent and men becoming concerned about being presumed sexist after witnessing gendered microaggressions.

To assess the processes by which witnessing gendered microaggressions affects enthusiasm for group work for men and women, stereotyping concerns were tested as mediators. General gender stereotyping concerns did not explain the effect of witnessing gendered microaggressions on enthusiasm for group work in Study 1 (although this effect did emerge for women in Studies 2a and 2b; see footnote 7 and online Supplementary Materials Section S8). Importantly, mediation processes were more fully examined in Studies 2a and 2b, in which gender-specific stereotyping concerns were tested. Specifically, for women, not men, witnessing gendered microaggressions decreased enthusiasm in part because it increased concerns about being presumed incompetent (Studies 2a and 2b). Consistent with social identity threat research (Steele et al., 2002), witnessing gendered microaggressions appears to serve as a stereotype cue that leads women to be concerned about being presumed by others to be incompetent in STEM, and may lead women to feel negatively about the prospect of working with a mixed-gender group. Such negativity could hinder successful collaboration and, instead, result in women engaging in more strained interactions, in which they may disengage from others in an attempt to avoid being stereotyped.

For men, mediation results were less consistent; in Study 2a, witnessing microaggressions decreased enthusiasm among men in part because it increased concerns about being presumed sexist, but this result was not replicated in Study 2b. Indeed, men’s concerns about being stereotyped as sexist were less affected, compared to women’s concerns about being stereotyped as incompetent, by witnessing gendered microaggressions. It may be that men are, overall, less concerned about being stereotyped, even in terms of being sexist, because they are positively stereotyped in STEM and are a privileged group in society, and their status protects them from feeling threatened by this social identity threat cue. Future work should continue to examine the effects of witnessing gendered microaggressions on men to increase our understanding of when and why men have negative reactions to this experience.

Although witnessing gendered microaggressions had strong, consistent effects across the studies on enthusiasm and stereotyping concerns, the effects on recall were only apparent in Study 1. In this in-person laboratory study, students who witnessed microaggressions among a group they expected to work with showed diminished recall of the STEM information presented by the group (compared to students who did not witness gendered microaggressions). In Studies 2a and 2b (online studies in which students did not expect to work with the group), this effect did not emerge. It may be that the effects on recall are more apparent when people witness gendered microaggressions in a group they believe they will soon actually work with (Study 1) than when they do not anticipate ever meeting the group (Studies 2a and 2b) because anticipation of working with the group is a more consequential experience. This possibility is consistent with work showing that experimental manipulations have greater effects on outcomes when they are experienced in “real life” as compared to when they are simply imagined (Clifford & Jerit, 2014; Finley & Penningroth, 2015).

### 4.1. Limitations and Future Directions

While this research has made significant contributions, several limitations should be acknowledged. First, our study adopted a gender-binary approach; that is, we examined how witnessing microaggressions among computer science and engineering students impacted both women and men, which is exclusive of individuals in computer science in engineering who fall outside the gender binary. Additionally, we did not examine how gender intersects with other social identities, such as race. For instance, prior self-report research in STEM workplace contexts finds that Black and Latina women’s competencies are highly likely to be questioned (compared to White and Asian American women), and they are frequently required to “prove” their competence in STEM (Williams et al., 2014). Thus, social identity cues that activate concerns about being stereotyped as incompetent could be particularly harmful to group work outcomes among Black and Latina women.

Furthermore, our focus was on short-term outcomes related to anticipating a group interaction, such as enthusiasm about working with the small group. However, prior research suggests that microaggressions are frequent, repeated occurrences, and negative effects on targets can accumulate over time (Sue, 2019; Hutchison, 2020). Future research should assess how repeated instances of simply witnessing microaggressions impact group dynamics and learning outcomes over time. For instance, a lack of enthusiasm for group work could result in disengagement from or avoidance of group work and missing out on the demonstrated benefits of team-based learning (Freeman et al., 2007; Haidet et al., 2014; Love et al., 2014). Addressing these limitations in future studies will enhance the robustness and applicability of the findings.

### 4.2. Contributions to Theory

The current research extends previous microaggression research because it took an experimental approach to examining the effects of witnessing microaggressions among men and women. Much previous research on microaggressions examined self-reported instances of being personally targeted by microaggressions. Although this previous work documented the prevalence of people experiencing microaggressions, and the significant consequences of this for people’s mental and physical health (Nadal et al., 2013; Wong et al., 2014; Wright & Lewis, 2020) and work outcomes (Hutchison, 2020; Kim & Meister, 2023; Yang & Carroll, 2018), the reliance on self-report has led to criticisms of microaggression research (e.g., Lilienfeld, 2017). Previous microaggression research typically focused on being the target of microaggressions, and our studies extend this work by focusing on merely witnessing microaggressions targeting other people. This is an important contribution to the extent that microaggressions occur in group contexts where they may be witnessed by others. Indeed, when a microaggression occurs in a group setting, there may be many witnesses (and only one target), suggesting that microaggressions may potentially affect more people as witnesses than as targets.

Our work also extended previous microaggression research by focusing on the effects of witnessing gendered microaggressions on not only women but men as well. There are few studies examining the effects of witnessing gender bias targeting women on male witnesses (see Pietri et al., 2019 for one example). Our findings highlight that witnessing microaggressions impacts all those in the setting by raising stereotyping concerns as well as diminishing enthusiasm about group work for both women and men.

### 4.3. Contributions to Practice

This work highlights the importance of interventions designed to reduce gendered microaggressions in educational settings such as universities, given their observed negative effect on men and women. Such interventions should be done using a multipronged approach, including increasing gender bias awareness, setting egalitarian social norms, and providing support to marginalized group members (Onyeador et al., 2021). Relatedly, prior research demonstrates that in male-dominated workplaces, support from female role models and mentors can reduce stereotype threat concerns and improve workplace satisfaction among women (Cortland & Kinias, 2019). This suggests that it is important to create opportunities for social support and mentorship for women in STEM settings to buffer against the negative effects of microaggressions. Our work also points out that microaggressions, which are, by definition, subtle, are by no means inconsequential. Although microaggressions are often perceived as minor, ambiguous, and harmless instances of bias (Sue, 2019), our research demonstrates that the negative effects of microaggressions extend beyond the individuals directly targeted. These effects may also impact everyone present in the environment who witnesses them.

## 5. Conclusions

The current research adds to our understanding of how people are affected by subtle gender bias in STEM settings. Although being targeted by gendered microaggressions is an important topic of study, this work focused on the consequences of merely witnessing gendered microaggressions on both women and men. Because there may be multiple witnesses to any single instance of subtle gender bias, witnessing it may be a common and prevalent experience. Understanding how witnessing gendered microaggressions harms all witnesses underscores the importance of reducing the prevalence of subtle gender bias behaviors in STEM settings, creating a more beneficial environment for everyone.

## Figures and Tables

**Table 2:** Summary of Studies 2a and 2b participants (note that due to rounding error, numbers do not add up to 100%).

	Study 2a	Study 2b
Initial Participants	412	272
Excluded Participants		
Failed attention checks (e.g., Please select somewhat disagree for this statement)	22	19
Indicated data of poor quality	37	–
Did not identify as current college students	46	–
Not engineering or computer science majors	27	–
Did not identify as men or women	–	10
Did not identify as US residents during last 5 years	–	3
Final number of participants	306	243
Country of Residence		
US	230	243
UK	72	0
Other	4	0
Gender		
Men	176	122
Women	130	121
Age		
Mean age	22.07 years (*SD* = 4.96)	22.00 years (*SD* = 6.13)
Race/Ethnicity		
White	52%	53%
Asian/Asian American	24%	24%
Black/African American	8%	6%
Latino/Hispanic	7%	5%
Middle Eastern or North African	1%	1%
Native American	<1%	<1%
Selected more than one race/ethnicity	5%	9%
Other	<1%	<1%

Note. Exclusion criteria are not mutually exclusive (i.e., a participant could be excluded for more than one reason).

**Table 3:** Gender-specific stereotyping concerns mixed ANOVA (Studies 2a and 2b).

	Study 2a		Study 2b	
	*F*(1, 302)	*p*	*F*(1, 239)	*p*
Condition	63.49	<0.001	28.48	<0.001
Gender	27.63	<0.001	16.85	<0.001
Stereotyping concern type	39.05	<0.001	18.40	<0.001
Condition × Gender	2.02	0.157	11.08	0.001
Gender × Stereotyping Concern	243.38	<0.001	244.23	<0.001
Condition × Stereotyping Concern	7.10	0.006	7.39	0.007
Condition × Gender × Stereotyping Concern	30.73	<0.001	29.24	<0.001

## Data Availability

Datasets and code underlying the findings presented are available on OSF: https://osf.io/zrhsn/?view_only=5ca599af01524daaae4af386b3f450b2, accessed on 24 January 2025.

## Notes

1 In each study, we over-recruited women to get a sizable sample of female computer science and engineering majors. In the online Supplementary Materials, Section S3, we report the number of men and women in the microaggression and control conditions for each study.

2 If two participants were scheduled for a lab session at the same time, they were told they would be joining separate groups, and one of them would go to another room.

3 Additionally, in each study, we included a manipulation check at the end to assess the participants’ perceptions of the videos as displaying respectful or biased interactions (see online Supplementary Materials, Section S7). In each study, the participants rated the neutral videos as more respectful and less gender-biased than the microaggression videos.

4 In the main text, we present results that were measured in each study and that we believe are important group work outcomes in the computer science and engineering context (engineering recall, stereotyping concerns, and enthusiasm for group work). Additionally, each study included exploratory control variables and moderators that did not significantly impact our interpretation of the results. Study 1 included exploratory outcomes that were not included in follow-up studies, and Study 2b was part of an omnibus data collection (of both male-dominated and gender-balanced STEM majors) and, thus, it included measures not of relevance to the current study. See the online Supplementary Materials, Section S10, for the full list of measures assessed in each study.

5 In Studies 2a and 2b, we again assess general gender stereotyping concerns. Results with the general gender stereotyping concern measure can be found in the online Supplementary Materials, Section S8.

6 Study 2b was part of a larger omnibus study and data collection effort involving both male-dominated and gender-balanced STEM majors (*N* = 568). In the current study, we only report on computer science and engineering college students (who identified as such in our internal survey).

7 In Studies 2a and 2b, we again measured general stereotyping concerns. Contrary to Study 1, both studies revealed a significant condition-by-gender interaction, with the effect of the condition on general stereotyping concerns being stronger for women. Moreover, both studies revealed a significant indirect effect of witnessing microaggressions on decreased enthusiasm through general stereotyping concerns for women, but not men (see online Supplementary Materials, Section S8 for full reporting of results). This further indicates that in small group work STEM contexts, stereotyping concerns may be of more relevance and more consequential to women than men.

## Supplementary Materials

The following supporting information can be downloaded at https://www.mdpi.com/article/10.3390/bs15020215/s1. Supplementary materials include secondary analyses, full statistical reporting, as well as texts of the experimental manipulation and survey items. A table of contents is included on the first page, detailing each section of the supplementary file. References (Morris, 2024) are cited in the supplementary materials.
