# Supplementary material for: Bystanders to Bias: Witnessing Gendered Microaggressions Affects Men’s and Women’s Outcomes in STEM Small Group Contexts
Source: Behav Sci (Basel). 2025 Feb 14;15(2):215. doi: 10.3390/bs15020215 (PMC11852149; doi:10.3390/bs15020215)
Supplement: Supplementary file 1 [file behavsci-15-00215-s001.zip › behavsci-3388388-supplementary.pdf]

The Supplement for:

"Bystanders to Bias: Witnessing Gendered Microaggressions Affects Men's and Women's Outcomes in STEM Small Group Contexts"

Table of Contents

Section S1. Description of Preliminary Survey **page 1**

Section S2. Description of Pretesting Results **page 6**

Section S3. Number of Participants per Cell **page 8**

Section S4. Full ANOVA Tables for Studies 1-2b **page 9**

Section S5. Means and Standard Deviations for Interactions Studies 2a & 2b **page 11**

Section S6. Correlation Tables for Studies 1-2b **page 12**

Section S7. Manipulation Check Results for Studies 1-2b **page 13**

Section S8. Study 2a and 2b General Stereotyping Concerns Results **page 14**

Section S9. Video Scripts and Survey Items **page 17**

Section S10. Full list of Measures for Omnibus Studies **page 22**

Section S1. Description of Preliminary Survey

In a preliminary survey we assessed whether STEM students more frequently reported witnessing compared to experiencing microaggressions and whether microaggression experiences were related to stereotyping concerns and sense belonging in one's field of study. A total of 201 STEM majors were recruited from Amazon's Mechanical Turk (133 men and 68 women) to participate in a survey that explores the experiences of college students in social and academic settings.

Measures

Witnessing and Experiencing Behaviors Implying Presumed Incompetence

Participants were presented with a series of descriptions of presumed incompetence and were asked whether they had experienced this themselves, witnessed a man experience this, or witnessed a woman experience this: "In this section, you will read about scenarios that students have reported while attending a university or other post-secondary educational program. You will be asked to rate the frequency with which you encounter these scenarios and the frequency with which you witness these scenarios happening to other people. It is important that you do not include your own experiences when making assessments of how often you see these situations happening to other people." Then participants read five scenarios, for example: "Someone assumed I was taking non-challenging classes because of my gender". Then participants were asked to indicate how frequently they had *witnessed men as targets* of presumed incompetence*,* ("I have seen this happen to a man", $\alpha = .92$), *witnessed women as targets* of presumed incompetence ("I have seen this happen to a woman", $\alpha = .89$), and *personally experienced* presumed incompetence ("This has happened to me," , $\alpha = .92$), each on a scale from 1 = Never to 5 = Very frequently.

Gender stereotyping concerns

Two items, adapted from Ramsey, Betz, and Sekaquaptewa (2013), were averaged together to assess participants concerns that one will be perceived in terms of gender stereotypes regarding their performances in their field of study (“I am concerned that others will judge people of my gender as a whole based on my performance in my field of study” and “I am concerned that people will think that my gender group as a whole has less ability if I do not do well in my field of study”; 1 = Strongly disagree to 5 = Strongly agree). The scale was scored so that higher numbers indicate greater gender stereotyping concerns (r = .81).

Belonging in field of study

Five items created by the authors were averaged together to assess participants’ perceptions that they belong in their field of study (e.g., “I feel like I really belong in my field of study”; 1 = Strongly disagree to 5 = Strongly agree). The scale was scored so that higher numbers indicate feeling higher levels of belonging in one’s field of study (α = .78).

Gender Based Rejection Sensitivity (individual difference variable)

Participants’ sensitivity to being rejected or discriminated against because of their gender was assessed with London et al.’s (2012) gender-based rejection sensitivity scale as a control variable. Items described various scenarios and for each scenario participants were asked to indicate how concerned they were that they would be rejected because of their gender and what they expected the outcome of the scenario to be (e.g., “Imagine that it's the first day of your science class and all the students must create teams to work on projects throughout the semester. Most of the groups are already full except for a few groups of all males. How concerned/anxious would you be that you might not be welcome to join one of the remaining groups because of your gender?” (1=Very unconcerned to 6=Very concerned); “I would expect to be welcome to join one of the remaining groups” (1=Very unlikely to 6=Very likely). In total there were 22-items which

were combined together according to London et al.'s (2012) scoring computation, such that a higher score indicates more gender-based rejection sensitivity (α = .91).

Results

Frequency of witnessing and experiencing presumed incompetence

Frequency of witnessing and experiencing presumed incompetence was analyzed using a 2 X 3 mixed model approach. Participant gender was entered as a fixed between-subjects factor (man, woman), the type of presumed incompetence was a fixed within-subjects factor (witnessing men as targets, witnessing women at targets, and personal experience), and their interaction term were entered. Responses to the type of presumed incompetence were nested within participants to account for the data structure, therefore participant ID was entered as a random factor (i.e., the intercept was allowed to vary). The outcome variable was the frequency of reporting presumed incompetence. GBRS was added as a control variable and therefore adjusted means are presented. This modeling approach was accomplished using the lme4 package in R, which does not provide p-value estimates due to the difficulties estimating p-values in mixed models, instead 95% confidence intervals of the coefficient estimate are provided (i.e., not including 0 indicates a significant effect). See Figure S1.1 for a graph of the results.

The mixed model revealed a fixed effect of gender *b* = -.90, *se* = .16, *t* = -5.72, 95% *CI*(-1.21, -.59), such that men reported a higher overall frequency of witnessing or personally experiencing presumed incompetence (M = 2.06, se = .07) than women did (M = 1.87, se = .09). There was also a fixed effect of target type b = .09, se = .03, t = -5.72, 95% CI(.03, .14). Pairwise comparisons revealed that people were most likely to report witnessing presumed incompetence directed at women (M = 2.24, se = .61) compared to witnessing presumed incompetence directed at men (M = 1.74, se = .61, t = -9.89, p < .0001) and compared to personally experiencing

presumed incompetence (M = 1.91, se = .61, t = 6.52, p < .0001). These effects were qualified by a two-way interaction, b = .36, se = .05, t = 6.36, 95% CI(.25, .47). Of primary interest was whether witnessing presumed incompetence directed at women was the most frequently reported among both men and women. Therefore, pairwise comparisons were applied comparing the frequency of each reported presumed incompetence type among men and comparing the types among women. Results revealed that witnessing presumed incompetence directed at women was the most commonly reported incompetence type among both women and men (see Figure S1.1).

Figure S1.1. Graph of presumed incompetence type by gender.

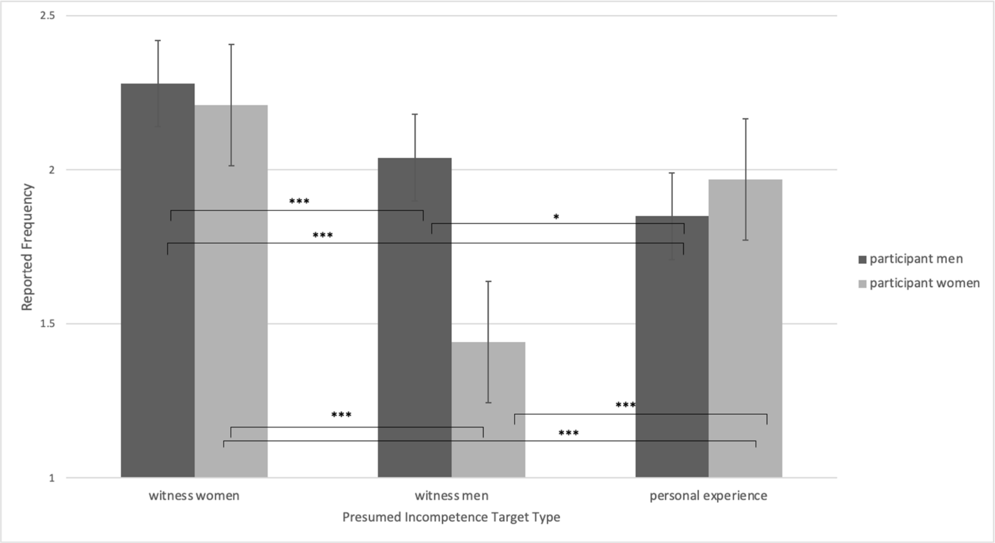

Note: 95% confidence intervals are represented with error bars. Plots are of adjusted means. Brackets with '*' represent significant pairwise comparisons. *p < .05, **p < .01, *** p <.001.

Presumed incompetence as a predictor of belonging and gender stereotyping concerns

A partial correlation analysis grouped by participant men and women was conducted to assess the relationship between each target type and gender stereotyping concerns and belonging in one's field. Gender-based rejection sensitivity was included as a control. See Table S1.1 below for correlation results. Of relevance to the focus of the current studies, the correlation analyses

show for both women and men participants witnessing presumed incompetence directed at women was related to higher levels of gender stereotyping concerns. For both genders witnessing presumed incompetence directed at women was not related to belonging.

Table S1.1. Correlations between types of presumed incompetence and belonging, and stereotype concerns by gender

	Women participants			Men participants		
	Personally experience	Witness women	Witness men	Personally experience	Witness women	Witness men
Belonging	-.06	-.03	-.27*	.12	.05	.07
Gender stereotyping concerns	49***	.33**	.22, p = .08	.32***	.29***	.32***

*p < .05, **p < .01, **p < .001

Discussion

The preliminary survey of college students indicated that both men and women students most often reported witnessing behaviors implying presumed incompetence directed at women, compared to presumed incompetence directed at men or directed at oneself. There was also no significant difference between men and women participants on the frequency with which they reported witnessing presumed incompetence toward women. Additionally, among both women and men witnessing presumed incompetence directed at women was related to increased stereotyping concerns (but not belonging with one's field of study). This provides initial evidence that it is important to study the effects of witnessing gender-based microaggressions directed at women on both men and women in STEM settings.

Section S2. Description of Pretesting Results

The research team created scripts depicting scenes of an engineering student team beginning to work on a project (Morris, 2024). Four student actors, 2 men and 2 women, were hired to portray the student engineering team in two scripted conditions: one including five events of gendered microaggression, and one with all neutral scenes (control). Each script included 9 scenes of which 4 were identical across the conditions as described in the text. The other 5 were each adapted as a microaggression and a neutral interaction (for the control condition).

Video clips of each of the scenes were produced and embedded into surveys for pre-testing to test whether the clips met the goals of demonstrating differences associated with the two conditions (i.e., being perceived as reflecting gender stereotyping or bias and being perceived as disrespectful or negative). Pretesting data was collected from 119 college students majoring in a STEM field (81 men, 38 women) who viewed and answered questions about the video clips in an on-line survey. Participants were randomly assigned to view five bias, or five control video-recorded group interactions, plus each group saw the four neutral filler interactions. Participants rated the video clips on the degree to which they reflected gender stereotyping or gender bias, as well as positive, respectful interactions on a 5-point Likert scale (strongly agree to strongly disagree). Pairwise comparisons were conducted to examine differences by condition as reported by the participants. Table S2.1 shows pre-test participant ratings of the levels of gender stereotyping or bias, and respect and positivity, for all nine scenes for both the microaggression and control video clips as well as the significance across condition.

Table S2.1 *Participant ratings and significance of of aspects of the videos*

Clip	Gender Stereotyping/Bias			Respectful/Positive		
	Micro	Ctrl	p-value	Micro	Ctrl	p-value

1	2.828	1.777	.001	3.013	3.982	<.001
2 (neutral)	1.651	1.896	NS	3.674	4.000	NS
3	2.849	2.108	.018	3.188	3.923	.007
4 (neutral)	1.719	1.980	NS	4.200	4.192	NS
5	2.849	1.837	.002	2.688	4.115	<.001
6	2.458	1.923	.079	3.363	3.872	.066
7 (neutral)	1.646	1.719	NS	4.125	4.3974	NS
8	3.094	1.899	<.001	2.713	4.385	<.001
9 (neutral)	1.932	1.953	NS	3.963	4.231	NS

Results showed that all the scenes containing microaggressions except scene 6 were found to be significantly higher in gender stereotyping/bias and significantly less respectful/positive than their control counterparts. Scene 6 was trending in this direction.

Full reference: Morris, D. L. (2024). Understanding subtle gender bias recognition among witnesses in a STEM undergraduate context. Open Access Dissertation, Michigan Technological University, 2024.

Section S3. Number of Participants per Cell

Here we report the number of men and women participants in the microaggression and control condition for each study.

Study 1

	**Microaggression**	**Control**
**Women participants**	54	52
**Men participants**	45	53

Study 2a

	**Microaggression**	**Control**
**Women participants**	68	62
**Men participants**	84	92

Study 2b

	**Microaggression**	**Control**
**Women participants**	55	66
**Men participants**	64	58

Section S4. Full ANOVA Tables for Studies 1-2b

In the following section we present the full ANOVA tables for the outcomes reported in the main text for each study.

Study 1

Engineering Recall

	F (1, 200)	p
Condition	9.18	.003
Gender	2.71	.102
Condition x Gender	.15	.699

Enthusiasm for group work

	F (1, 200)	p
Condition	40.04	< .001
Gender	1.33	.250
Condition x Gender	.04	.834

Stereotyping concerns

	F (1, 200)	p
Condition	13.09	< .001
Gender	128.05	< .001
Condition x Gender	1.39	.241

Study 2a

Engineering Recall

	F (1, 302)	p
Condition	.49	.485
Gender	.01	.941
Condition x Gender	.06	.809

Enthusiasm for group work

	F (1, 302)	p

Condition	92.85	< .001
Gender	.21	.650
Condition x Gender	3.49	.063

Study 2b
Engineering Recall

	F (1, 239)	p
Condition	.06	.813
Gender	.38	.537
Condition x Gender	.96	.327

Enthusiasm for group work

	F (1, 302)	p
Condition	148.10	< .001
Gender	.23	.631
Condition x Gender	15.22	< .001

Note. We do not report full ANOVA tables for the gender specific-stereotyping concerns in Studies 2a and 2b because this is already reported in the main text.

Section S5. Means and Standard Deviations for Interactions Studies 2a & 2b

In this section we report the means and standard deviations for significant interactions from Studies 1 - 2b (means and 95% Confidence Intervals are displayed graphically in the main text)

Study 2b Mean and Standard Deviations by Condition and Gender for Enthusiasm about Group Work

	Microaggression	Control
Men	3.30 (1.60)	4.71 (1.37)
Women	2.56 (1.12)	5.29 (1.12)

Study 2a Mean and Standard Deviations by Condition and Gender for Assumed Sexist Stereotyping Concern

	Microaggression	Control
Men	3.39 (1.98)	2.24 (1.48)
Women	2.56 (1.20)	1.94 (.89)

Study 2a Mean and Standard Deviations by Condition and Gender for Assumed Incompetent Stereotyping Concern

	Microaggression	Control
Men	2.38 (1.36)	1.68 (.91)
Women	5.09 (1.50)	3.07 (1.63)

Study 2b Mean and Standard Deviations by Condition and Gender for Assumed Sexist Stereotyping Concern

	Microaggression	Control
Men	3.89 (1.82)	3.28 (1.73)
Women	2.81 (1.30)	2.31 (1.06)

Study 2b Mean and Standard Deviations by Condition and Gender for Assumed Incompetent Stereotyping Concern

	Microaggression	Control
Men	2.39 (1.27)	2.36 (1.36)
Women	5.82 (1.19)	3.58 (1.87)

Section S6. Correlation Tables for Studies 1-2b

In the following section we report correlations among the outcomes reported in the main text for each study. Of relevance to our mediation predictions, correlations indicate stereotyping concerns are related to enthusiasm for group work, but not related to recall of engineering information.

Study 1

	Engineering recall	Enthusiasm for group work	Stereotyping concerns
Engineering recall	-		
Enthusiasm for group work	.10, p = .151	-	
Stereotyping concerns	-.07, p = .312	-.16, p = .025	-

Study 2a

	Engineering recall	Enthusiasm for group work	Stereotype concern sexist	Stereotype concern unskilled
Engineering recall	-			
Enthusiasm for group work	.07, p = .196	-		
Stereotype concern sexist	.02, p = .766	-.25, p < .001	-	
Stereotype concern unskilled	-.03, p = .648	-.35,p < .001	.32, p < .001	-

Study 2b

	Engineering recall	Enthusiasm for group work	Stereotype concern sexist	Stereotype concern unskilled
Engineering recall	-			
Enthusiasm for group work	.06, p = .373	-		
Stereotype concern sexist	-.12, p = .057	-.19, p = .003	-	
Stereotype concern unskilled	-.10, p = .122	-.30, p < .001	.11, p = .090	-

Section S7. Manipulation check results for Studies 1-2b

In all three studies we included a manipulation check to ensure that the control condition was perceived as more respectful (Studies 1- Studies 2b) and containing less gender bias (Studies 1 - Studies 2a) than the microaggression condition. As expected, in line with pre-testing, this was the case.

In Study 1, the control condition was perceived to be more respectful (M = 3.78, SD = .79) than the microaggression condition (M = 2.37, SD = .81) (t = -12.58, p < .001), and the control condition was perceived to contain less gender bias (M = 2.30, SD = .98) than the microaggression condition (M = 3.64, SD = 1.01) (t = 9.53, p < .001).

In Study 2a, the control condition was also perceived to be more respectful (M = 4.25, SD = .71) than the microaggression condition (M = 2.89, SD = 1.02) (t = -13.57, p < .001), and the control condition was perceived to contain less gender bias (M = 1.65, SD = .84) than the microaggression condition (M = 3.32, SD = 1.26) (t = 13.70, p < .001).

Lastly, in Study 2b, the control condition was also perceived to be more respectful (M = 5.92, SD = 1.02) than the microaggression condition (M = 3.49, SD = 1.47) (t = -14.93, p < .001) (perceptions of gender bias were not measured).

Section S8. Study 2a and 2b General Stereotyping Concerns

In Studies 2a and 2b we also measured general stereotyping concerns in addition to gender specific stereotyping concerns. Participants answered the same 2-items about general stereotyping concerns as they did in Study 1. Below we report the 2 (control, microaggression) X 2 (men, women) ANOVA results and multigroup path analysis results with the general stereotyping concern measure.

ANOVA Results

Study 2a. In Study 2a results revealed a main effect of condition and gender qualified by a significant two-way interaction (see Table below). Simple main effects indicated the effect of condition was significant for men ($F(1, 174) = 6.91$, $p = .009$), such that men participants in the microaggression condition reported greater stereotyping concerns ($M = 2.58$, $SD = 1.56$) than those in the control condition ($M = 2.01$, $SD = 1.36$). Simple main effects also indicated a larger effect of condition for women participants ($F(1, 128) = 57.96$, $p < .001$), such that women (more so than men) reported greater stereotyping concerns in the microaggression condition ($M = 4.95$, $SD = 1.60$) compared to the control condition ($M = 2.82$, $SD = 1.58$).

General Stereotyping Concerns S2a ANOVA Results

	F (1, 302)	p
Condition	59.36	< .001
Gender	82.23	< .001
Condition x Gender	19.46	< .001

Study 2b. In Study 2b results also revealed a main effect of condition and gender qualified by a significant two-way interaction (see Table below). Simple main effects indicated the effect of condition was not significant for men ($F(1,120) = 1.23$, $p = .27$), such that men participants in the

microaggression condition did not report statistically significantly greater stereotyping concerns (M = 2.57, SD = 1.57) than those in the control condition (M = 2.27, SD = 1.48). However, simply main effects did reveal a significant effect of condition for women participants (F(1, 119) = 16.07, p < .001), such that women (more so than men) reported greater stereotyping concerns in the microaggression condition (M = 5.22, SD = 1.38) compared to the control condition (M = 4.01, SD = 1.85).

General Stereotyping Concerns S2b ANOVA Results

	F (1, 239)	p
Condition	13.81	< .001
Gender	116.03	< .001
Condition x Gender	4.96	.027

Multigroup Path Analysis

Study 2a. In Study 2a path analysis results indicate that for women participants an indirect effect, such that the effect of witnessing microaggressions on diminished enthusiasm is in part explained by increased general stereotyping concerns. However, the indirect effect is not significant for men participants (see Figure below).

Figure of Path Analysis in Study 2a

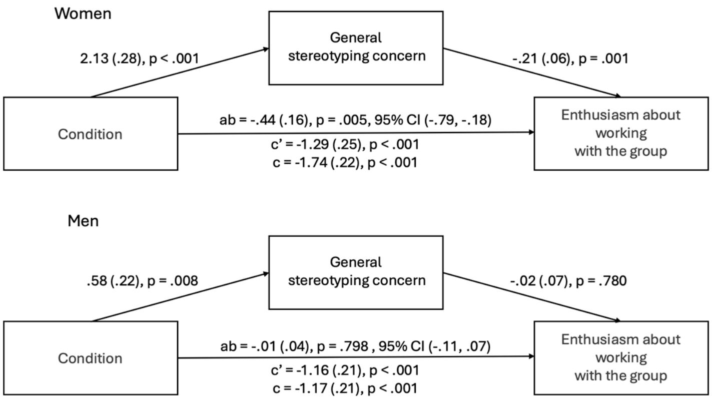

Study 2b. Study 2b revealed the same pattern of results such that the indirect effect was significant for women participants, but not men participants (see Figure below). Thus, indicating that the activation of stereotyping concerns from witnessing microaggressions in particular is damaging for women's peer interactions.

Figure of Path Analysis in Study 2b

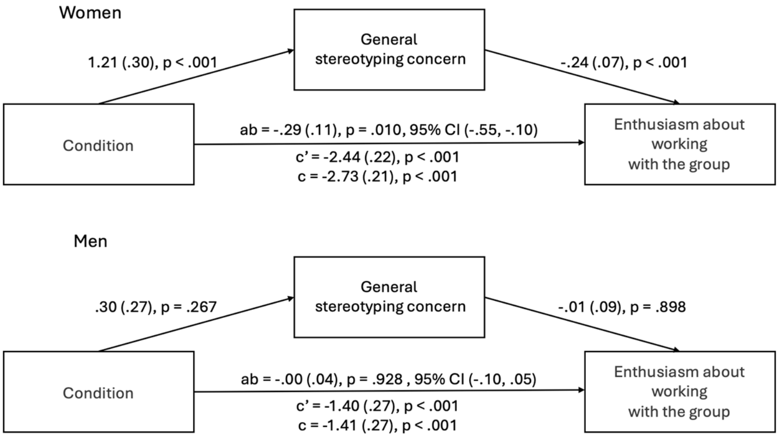

Section S9. Video script and survey items

In the following section we present the transcript of the video interactions presented in all three studies, and we also present the survey items that constitute the measures reported in the main text for each study.

Table S8.1 *Video scripts for each scene with differences for the microaggression and control scenes underlined.*

Scene	Microaggression	Control
1	**Ethan**: Cool, so at our last meeting we established the timeline. Now based on that we need to create a design for the rip current sensor, so let's get started. So someone needs to take notes.<br>**Rachel (hesitantly)**: I can do it.<br>**Ethan**: I'd do it but my handwriting is so bad.	**Ethan**: Cool, so at our last meeting we established a timeline and now based on that we have to create a design for the rip current sensor so let's get started. So someone needs to take notes.<br>**Ryan**: I can do it.<br>**Ethan**: Thanks.
2 (Neutral)	**Erika**: Hey, do any of you remember if we're allowed to look at the camera during this, because I've definitely made eye contact with it on a couple occasions.<br>**Ryan**: I keep forgetting we're being recorded.	

	**Ethan**: A couple of my friends have done studies. They say the researchers are pretty laid back.<br>**Rachel**: It probably doesn't matter as long as we don't all stare at the same time. I'm pretty sure they only care about what we talk about in the meeting. Speaking of which, what do we need to talk about?	
3	**Ethan**: Okay let's start with what color the buoy should be. What do you all think?<br>**Erika**: What's a good visible color?<br>**Rachel**: Neon orange? How about neon orange?<br>**Ethan**: Day-glow green?<br>**Erika**: What if there's like an algae bloom where the water turns all green?<br>**Ryan**: Right, yeah, if the water was bright green like that then you wouldn't see it, so it has to be a color that water never is.<br>**Ethan**: Okay yeah, yeah I grew up on a lake, that does happen water gets super green.<br>**Ryan**: Um... well you know how construction workers wear those neon orange reflective vests?<br>**Ethan**: Yeah, neon orange - that works. Good one, Ryan!	**Ethan**: Okay let's start with what color the buoy should be. What do you all think?<br>**Erika**: What's a good visible color?<br>**Rachel**: Neon orange? how about neon orange?<br>**Ethan**: Day glow green?<br>**Erika**: What if there's like an algae bloom where the water turns all green?<br>**Ryan**: Right, yeah, if the water was bright green like that then you wouldn't see it, so it has to be a color that water never is.<br>**Ethan**: Okay yeah, yeah I grew up on a lake, that does happen water gets super green. Yeah, let's just go with what Rachel said.<br>**Ryan**: That works.<br>**Ethan**: Neon orange it is.
4 (neutral)	**Erika**: So what lake did you grow up on?<br>**Ethan**: Oh, my family owns a cabin up north on Bear Lake. We go up there every summer and weekends and stuff.<br>**Erika**: That's cool we used to go to the UP every summer for a few weeks and room to cabin on the lake.<br>**Ethan**: Nice.	
5	**Ryan**: Okay... so now don't have to have something that'll make it launchable from like 300 feet?<br>**All**: Yeah<br>**Ryan**: What about a t-shirt gun? Would a t-shirt gun shoot that far?<br>**Erika**: Where are we going to get—<br>**Ethan (interrupting)**: Yeah they definitely shoot 300 feet.<br>**Erika**: Okay well I guess you could call---	**Ryan**: Okay... so now don't have to have something that'll make it launchable from like 300 feet?<br>**All**: Yeah<br>**Ryan**: What about a t-shirt gun? Would a t-shirt gun shoot that far?<br>**Erika**: Where are we going to get that? Can you buy one of those at the store?<br>**Ethan**: Not sure where you buy one, but they definitely go 300 feet.

	**Ethan (interrupting)**: You'd have to have like a lot of CO2 to make it go 300 feet.<br>**Rachel**: Let's just research it.	**Erika**: Maybe we can call a sports stadium and see where they get them?<br>**Ryan**: You know, I bet you need to use a lot of CO2 to make it shoot 300 feet.<br>**Rachel**: Let's just research it.
6	**Ryan**: Uh... so what do you guys think about Calc 2? Super hard right?<br>**Erika**: I didn't think it was so bad, just a lot of problem sets. So Rachel, what section of calc 2 are you in?<br>**Rachel**: Oh, I'm not in Calc 2, I'm in Calc 3 with Professor Montgomery.<br>**Ryan (looking surprised)**: Seriously, you're in Calc 3? That's amazing!	**Ryan**: Uh... so what do you guys think about Calc 2? Super hard right?<br>**Erika**: didn't think it was so bad, just a lot of problem sets. So Rachel, what section of calc 2 are you in?<br>**Rachel**: Oh I'm not in Calc 2, I'm in Calc 3 with Professor Montgomery.<br>**Ryan**: Cool.
7 (neutral)	**Rachel**: Hey, check this out, you can build your own t-shirt cannon.<br>**Ethan**: Oh! It costs less than $100 too.<br>**Erika**: Well that might be a good option rather than buying one. The cheapest one I can find is $300.<br>**Ryan**: Does it give you instructions on the website?<br>**Rachel**: Yeah, I just downloaded a pdf, we can print out later.<br>**Erika**: Great, what's next?	
8	**Erika**: So, going back to launching it, I actually have another idea: what about using a remote control boat to pull it into position? I've seen it a lot, even pulling water skiers and it might be more accurate.<br>**Ryan**: That's a neat idea but do we have enough money for an RC boat?<br>**Ethan**: Are you familiar with opportunity cost?<br>**Erika**: Yeah, I took an econ class that covered it. An---<br>**Ethan (Interrupting)**: Well it's basically like-- it's like if you choose one alternative, then you're giving something up by not choosing the other.<br>**Erika**: Yeah I know what---<br>**Ethan (Ignoring)**: So since our project is about saving lives in the rip current, we have to choose something that's super reliable and then we'll weigh the cost of	**Erika**: So going back to launching it, I actually have another idea: what about using a remote control boat to pull it into position? I've seen them a lot, even pulling water skiers, and it might be more accurate.<br>**Ryan**: That's a neat idea, but do we have enough money for an RC boat?<br>**Ethan**: Are you familiar with opportunity cost?<br>**Erika**: Yeah, I took an econ class that covered it. An opportunity cost is when you pick one option you're automatically giving up something else by picking up the other options.<br>**Ethan**: Right, yeah, exactly. And, so, since our project is about saving lives in the rip current, we have to choose something that's super reliable and so then we'll weigh the cost of the RC boat and the other ideas, like the t-shirt launcher against the reliability.

	the RC boat and the other ideas, like the t-shirt launcher against the reliability.<br>**Ryan**: Cool.<br>**Ethan**: Yeah.	**Ryan**: Cool.<br>**Ethan**: Yeah.
9<br>(neutral)	**Ryan**: Real quick – could we look at the handout they gave us for the project?<br>**Erika**: Oh sure I've got it on the drive, let me pull it up.<br>**Ryan**: Thanks.<br>**Erika**: So what did you want to look at?<br>**Ryan**: I just want to look at the specs again, make sure we're headed in the right direction on the design.<br>**Erika**: Okay so here's what it says: the buoy should hold the GPS tracker face up above the water; the buoy should be launchable from the shore out to a distance of 300 feet; the buoy should have a device hanging beneath it that is designed to catch the current and move the device with the current; the buoy should be brightly colored so it can be seen from a distance of 500 feet.<br>**Ryan**: Right, okay, we still need to do something about the device hanging beneath it.	

Full wording of survey items

Study 1
Enthusiasm for group work
1 - Strongly disagree to 7 - Strongly agree
I wish I could join a different group than the one I was assigned to.
I am looking forward to working with this group.
I feel positive about the idea of working with this group.
1 - Strongly disagree to 7 - Strongly agree

Stereotyping concerns
1 - Strongly disagree to 7 - Strongly agree
I am concerned that others will judge people of my gender as a whole based on my performance in the group task.
Others will think that people of my gender as a whole have less ability if I do not do well in the group task.

Engineering recall
1. What is the purpose of the buoy being designed?
    a. To measure rip currents
    b. To measure flow in a river
    c. To measure boat speed
    d. To mark a swimming area

2. How far should the buoy be launched from shore?

a. 100 ft
b. 200 ft
c. 300 ft
d. 400 ft

3. How should the buoy hold the GPS tracker?
    a. Face up above the water surface
    b. Face down above the water surface
    c. Face up below the water surface
    d. Face down below the water surface

4. From how far away should the buoy be visible?
    a. 200 ft
    b. 300 ft
    c. 400 ft
    d. 500 ft

5. The device should be designed to move:
    a. Against the current
    b. With the current
    c. Tangential to the current
    d. The device shouldn't move, it should stay in position

Study 2a
(instructions) Imagine that you were going to join the group you just saw to work on a similar task.

Enthusiasm for group work
Please indicate how much you agree or disagree that you would feel the following...
1 - Strongly disagree to 7 - Strongly agree
I would want to join a different group than the one I was assigned to.
I would look forward to working with this group.
I would feel positive about the idea of working with this group.

General stereotyping concern
1- Not at all to 7 - Very much
How concerned would you be that others will judge people of your gender as a whole based on your performance in the group task.
How much do you think other people will think that people of your gender have less ability if you do not do well in the group task.

Stereotyping concern incompetent
1- Strongly disagree to 7- Strongly agree
I am concerned that other people in the group will assume people of my gender are incompetent/not

smart.
I am concerned that other people in the group will assume people of my gender are unskilled in science and engineering.
I am concerned that other people in the group will assume people of my gender do not possess the skills to perform well on the group task.

Stereotyping concern sexist
1- Strongly disagree to 7- Strongly agree
I am concerned that other people in the group will likely assume people of my gender are sexist.
I am concerned that other people in the group will think people of my gender are biased against women [men].

Engineering recall
Same as Study 1

Study 2b
Same items as Study 2a

Section 10. Full list of Measures for Studies

In the tables below we present the full list of measures asked in each study. *In the current research, we focus on presenting results that were measured in each study and that we believe are important group work outcomes in the computer science and engineering context (engineering recall, stereotyping concerns, and enthusiasm for group work).* A manipulation check was also measured in each study and is presented in the Supplement, Section 7.

Additionally, each study included exploratory moderators and control variables which did not significantly impact the interpretation of results, and thus we do not focus on and we did not measure consistently across studies (e.g., gender based rejection sensitivity in Study 1, ambivalent sexism in Study 2a, negative emotionality in Study 2b). Study 1 also included some exploratory outcomes (e.g., spatial ability, stereotype activation word completion) that were not included in Studies 2a and 2b.

Lastly, Study 2b was part of an omnibus data collection of participants in gender balanced and male-dominated STEM fields. For cost and recruitment feasibility we included our measures of interest as part of this data collection to provide an opportunity for replication of Study 2a. Thus,

Study 2b also included measures that are the focus of a different project (e.g., stereotype endorsement). In the current research, we only report on computer science and engineering participants and the outcomes of focus in the current project (consistent with Studies 1 and 2a).

Study 1 Full List of Measures

Informed consent
Manipulation (video)
Stereotype activation word completion task
Spatial ability test
**Engineering recall**
**General stereotyping concern**
**Enthusiasm for group work**
Performance expectations and motivation
Identification with team
Belonging with field of study
Gender based rejection sensitivity
**Manipulation check**
Demographics
Debrief

Study 2a Full List of Measures

Informed consent
Confirm major is computer science or engineering
Perceptions of success in field of study
Identification with field
Identification with gender
Conscientiousness
Manipulation (video)

Open ended prompt (recall information presented in the videos)
**Engineering recall**
**Enthusiasm for group work**
**Stereotype threat concerns (general and gender specific)**
**Manipulation check**
Ambivalent sexism
Sensitivity to gender bias
Demographics
Debrief

Study 2b Full List of Measures

Informed consent
Confirm major is computer science or engineering
Perceptions of success in field of study
Manipulation (video)
Open ended prompt (recall information presented in the video)
**Engineering recall**
Stereotype endorsement measure
Science and Gender IAT
**Enthusiasm for group work**
**Stereotype threat concerns (general and gender specific)**
Sensitivity to gender bias
Negative emotionality measure
Demographics
**Manipulation check**
Debrief
